# The temporal progression of retinal degeneration and early-stage idebenone treatment in the Pde6b^rd1/rd1^ mouse model of retinal dystrophy

**DOI:** 10.1038/s41598-024-52391-y

**Published:** 2024-01-23

**Authors:** Lei Zhang, Wei Liu, Hai-Yan Wang, Wei Qiang, Ru Wang, Zhi-Li Cui, Zuo-Ming Zhang

**Affiliations:** 1 Shaanxi Eye Hospital, Xi’an People’s Hospital (Xi’an Fourth Hospital), Xi’an, 710004 China; 2 Xi’an Key Laboratory of Digital Medical Technology of Ophthalmologic Imaging, Xi’an People’s Hospital (Xi’an Fourth Hospital), Xi’an, 710004 China; 3 Medical Experiment Center, Shaanxi University of Chinese Medicine, Xianyang, Shaanxi Province China; 4 Department of Clinical Medicine, Faculty of Aerospace Medicine, Air Force Medical University, Xi’an, 710032 Shaanxi Province China

**Keywords:** Experimental models of disease, Disease model, Hereditary eye disease, Retinal diseases

## Abstract

Photoreceptor cell death, primarily through apoptosis, related to retinal disorders like retinitis pigmentosa (RP), would result in vision loss. The pathological processes and crucial mutant conditions preceding photoreceptor cell demise are not well understood. This study aims to conduct an in-depth examination of early-stage changes in the widely utilized Pde6b^rd1/rd1^ (rd1) mouse model, which has *Pde6b* gene mutations representing autosomal recessive RP disorder. We investigated the morphology and ultrastructure of retinal cells, including second-order neurons, during the initial phase of disease progression. Our findings revealed that mitochondrial alterations in rod photoreceptors were present as a predeath mutant state as early as postnatal day 3 (P3). The bipolar and horizontal cells from the rd1 mouse retina exhibited significant morphological changes in response to loss of photoreceptor cells, indicating that second-order neurons rely on these cells for their structures. Subsequent oral administration of idebenone, a mitochondria-protective agent, enhanced retinal function and promoted both photoreceptor cell survival and inner retinal second-order synaptogenesis in rd1 mice at P14. Our findings offer a mechanistic framework, suggesting that mitochondrial damage acts as an early driver for photoreceptor cell death in retinal degeneration.

## Introduction

RP (Retinitis Pigmentosa) is a leading global cause of irreversible vision loss. This condition is generally attributed to inherited genetic mutations primarily detected in either retinal pigment epithelial (RPE) cells or photoreceptor cells^1^. Currently, no effective therapies exist that can halt or reverse the progressive degeneration of photoreceptors or RPE loss among those affected by RP. Consequently, examining the pathophysiological alterations, especially during the initial stages of RP, and devising treatment approaches focused on inhibiting its progression, are of paramount importance.

Spontaneous mutations in mice, as well as mutations induced in mouse models, effectively mimic the retinal diseases observed in human patients^2^. A common mutation observed spontaneously in mice is the retinal degeneration 1 (rd1) mutation. This mutation is recessive and harbors a nonsense mutation in exon 7 of the *pde6b* gene^3^. This mutation disrupts the gene responsible for encoding the rod β-subunit of the cyclic guanosine monophosphate (cGMP) phosphodiesterase (PDE), leading to retinal degeneration^3^. Mutations in the PDE6B gene in humans lead to various phenotypes of autosomal recessive retinal degeneration^4^. Mice carrying the homozygous rd1 mutation prenatally develop photoreceptors normally^5^. However, these mice lose all rod photoreceptors within just two weeks post-birth and subsequently experience a progressive decline in cone photoreceptors^5^.

Although discovered nearly a century ago, most studies have concentrated on phases either during or after degenerative onset, or at advanced stages following significant photoreceptor loss^5–7^. At present, retinal remodeling secondary to photoreceptor degeneration has been proved to occur in rd1 mice and in other animal models of retinal degeneration^8–13^. This occurred in human RP retinas as well^8,14,15^. The remodeling was vulnerable to photoreceptor degeneration and began at the time of synaptogenesis^9^. Major neurochemical remodeling including glutamine and glutamate occurred prior to anatomical remodeling^10,16,17^. In addition, existing neurons sprouted abnormal processes termed “rewiring”^8,18,19^. Phenotypic switching on existing cells resulted in “reprogramming” of neural circuits^20^. The cell death of many neurons of all types within the inner retina was the final effect. However, the pathological processes that precede photoreceptor cell death and the initiators of remodeling remain largely unexplored. Fukuda et al. showed that oxidative stress retarded vascular development before neural degeneration occurred only in rd1 mice, not all the rodent models^21^. To decide the suitable therapeutic windows, prevention of remodeling may have to precede intervention. Therefore, it is imperative to investigate the initial pathological changes of retinal degeneration to look for the earliest common regulatory pathways.

In prior research, we identified a Kunming mouse strain with spontaneous RP and studied its retinal tissue morphology starting from postnatal day 14 (P14)^22^. The outer nuclear layer of the mutated mice was significantly reduced at P14 and contained a nonsense mutation in exon 7 of the *pde6b* gene^22^. According to the ocular phenotype and genotype, it was identified as rd1^22^. In the current study, we employ this well-studied retinal neurodegeneration model—derived from a cross between C57BL/6 J and the mutated Chinese Kunming mouse strain—to explore the molecular and cellular events occurring before P14 that may lead to photoreceptor cell death. The goal of this study is to offer a comprehensive analysis of the morphology and ultrastructure of retinal cells, including second-order neurons, in the early developmental stages of the rd1 mouse retina. Additionally, we strive to identify a common degenerative pathway that operates independently of specific gene mutations. Our research could illuminate new approaches for potentially slowing down photoreceptor cell degeneration. Even in the absence of a complete cure, these insights could substantially enhance the quality of life for individuals with RP, extending their capacity for independent living.

## Methods

### Ethical statement

All experimental procedures were approved by the animal care and use committee at Xi’an People’s Hospital, also known as Xi’an Fourth Hospital. The study followed ARRIVE guidelines (https://arriveguidelines.org).

### Animals

Mice were housed in a 12-h light–dark cycle environment. Pde6b^rd1/rd1^ mice, commonly referred to as rd1, were obtained from Dr. Zuo-Ming Zhang's laboratory, which is located at the Center of Clinical Aerospace Medicine, Air Force Medical University in Xi'an, Shaanxi Province, China. The *pde6b* gene's nonsense mutation in rd1 mice was verified as described earlier^22^. In all experiments, C57BL/6 J mice served as wild type (WT) controls, and the research incorporated both male and female mice. All the mice used for experiments were anesthetized through an intraperitoneal administration of 1% sodium pentobarbital and Sumianxin II and sacrificed with carbon dioxide after the use.

### Idebenone treatment

Idebenone powder was sourced from Qilu Pharmaceutical Co., Ltd. (Shandong, China). Based on prior research^23^, a dose of 200 mg/kg of idebenone was given to lactating mother mice through a single daily gavage, wherein the idebenone was dissolved in PBS at a concentration of 20 mg/ml in water. This treatment was administered from postnatal day 0 to 21.

### Retinal histology

Enucleated eyes from euthanized rd1 mice of various postnatal ages were submerged in 4% glutaraldehyde at room temperature for 30 min. Following the removal of the cornea, lens, and vitreous body, the eyecup was fixed in 4% paraformaldehyde at 4 ℃ overnight. The tissue samples were subsequently embedded in paraffin and serial sections with a 3 μm thickness were prepared. For each eye, three sections were selected with the optic nerve head (ONH) as a reference point and stained with hematoxylin and eosin (HE).

### Thickness assessment of the total retinal layer, retinal inner nuclear layer (INL) and outer nuclear layer (ONL)

A digital imaging system (SlideViewer2.5, PannoramicMIDI, 3DHISTEC, Hungary) was utilized to capture images, which were subsequently analyzed by determining the total retinal thickness (TRT, ranging from the retinal nerve fiber layer to the RPE) and counting the number of rows of INL and ONL. Thickness of retinal layers were measured on the HE images at specific locations from the ONH. Measurement results were plotted in a spider graph. At least 3 eyes (from 3 different mice) per group were used in the statistical analysis.

### Transmission electron microscopy examination

Eyecup retinal samples were fixed using a solution containing 1% paraformaldehyde and 2.5% glutaraldehyde in phosphate-buffered saline (PBS) with a pH of 7.4. The samples were then treated with 1% osmium tetroxide. Fixed specimens underwent dehydration through a series of ethanol gradients and propylene oxide and then were embedded in Epoxy resin 812. Sections of the retina samples, ranging from 60 to 90 nm in thickness, were placed on copper grids for further analysis. Uranyl acetate and lead citrate were applied to stain the sections, which were then imaged with a JEM-1400FLASH (JEOL, Japan) transmission electron microscope. To quantify the different states of mitochondria and the Golgi apparatus in the developing rd1 mice (ultrastructural analysis), 3 eyes from 3 different mice was used per time point. 6 random fields per eye were taken for the inner segment region of the photoreceptors at 25,000× magnifications. To quantify the different states of ribbon, 6 random fields per eye (3 eyes from 3 different mice at P14) were taken for the outer plexiform layer (OPL) at 25,000× magnifications. (N = 3 eyes from 3 different mice per group, n = 6 images from each eye). The proportion of abnormal mitochondria (abnormal/total mitochondria), the number of Golgi apparatus and ribbon per field were measured.

### RNA extraction and qRT-PCR

Takara RNAiso Plus reagent (D9109, Takara, Japan) was used to extract total RNA from retinal tissue. By spectrophotometry (Nanodrop 2000; Thermo Scientific, Waltham, MA, USA), total RNA concentration was determined. Using a PrimeScript RT Master Mix Kit (RR037A; Takara, Shiga, Japan), cDNA was synthesized from total RNA. Real-time PCR was performed on an Applied Biosystems 7500 Real-Time PCR System (Applied Biosystems, Foster City, CA, USA) using a SYBR Green premix EX Taq II kit (RR820A; Takara). Genes were normalized to the *β-actin* value. The expression level of *pde6b* gene was calculated by the 2^−ΔΔCt^ method. The primer sequences were: *β-actin* (F) GCT GTG CTA TGT TGC TCT AG and (R) CCA AGA AGG AAG GCT GGA; *pde6b* (F) CAG CAT GAA CAT GTG ATC CA and (R) TCG TGT GGT CTC TAA GGA TAA G.

### Electroretinogram (ERG) and flash visual-evoked potentials (F-VEP)

Following overnight dark adaptation, mice were thoroughly anesthetized through an intraperitoneal administration of 1% sodium pentobarbital and Sumianxin II. Their pupils underwent dilation using a 0.5% tropicamide-phenylephrine ophthalmic solution. For ERG recordings, a silver-chloride electrode loop encased in a layer of 1% methylcellulose, was placed on the cornea, and referenced to a needle electrode in the mouth. A needle electrode placed in the tail served as the ground. After ERG recording, the F-VEP was performed. The active electrode for the F-VEP (a steel needle) was placed in the middle of two ears. The left eye (not stimulated) was occluded with a dark patch for VEP recording. Full-field (Ganzfeld) stimulation and a computation system (RETI port, Roland Consult GmbH, Brandenburg, Germany) were utilized. Rod ERG, Maximum scotopic ERG, Cone ERG, and F-VEP were recorded in accordance with the International Society for Clinical Electrophysiology of Vision (ISCEV) guidelines^24,25^.

### Immunohistochemical and immunofluorescent staining

Retinal tissue sections were prepared as described in the retinal histology protocol. The sections underwent dewaxing with xylene and were subsequently rehydrated through a range of ethanol concentrations. To inhibit endogenous peroxidase activity, the sections were treated with 3% hydrogen peroxide for a duration of 20 min. For antigen retrieval, the sections were submerged in a citrate buffer solution (pH = 6.0) and exposed to microwave heating in three separate 5-min intervals. Once the sections reached room temperature, they were washed with PBS three times, each for 10 min. A 5% bovine serum albumin (BSA) (BioFROXX, Germany) solution was then used to block the sections for an hour before proceeding with immunohistochemical or immunofluorescent staining. Sections containing the optic nerve were specifically chosen for immunohistochemistry and were incubated with primary antibodies (Table 1) at 4 ℃ overnight.

**Table 1:** Antibodies utilized for immunohistochemical and immunofluorescent staining.

Antibody	Company	Dilution (IHF/IHC)	Host	Cat#
CtBP2	Novus	1:50/1:200	Rabbit	NBP3-15268
Calbindin D28K	Novus	1:1000/1:1000	Chicken	NBP2-50028
PKCα	Novus	1:100/1:600	Mouse	NB600-201

*IHF* immunofluorescent staining, *IHC* immunohistochemical staining.

For single-signal labeling, immunohistochemical staining was used. Following five washes with PBS, the sections underwent incubation with a biotinylated secondary antibody and streptavidin peroxidase (KIT9730, MXB, China) for a duration of 50 min at a temperature of 37 ℃. DAB (DAB-1031, MXB, China) served as a chromogen. Images of immunohistochemical staining were captured using a SlideViewer2.5 system (PannoramicMIDI, 3DHISTEC, Hungary). For the semi-quantification of immunohistochemical staining, three eyes (from 3 different mice) in each group were selected, and 5 randomly selected fields in the retinas were captured (40×) at P7 and P14. ImagePro Plus 6.0 software was used to calculate the integral absorbance and area of positive results under each field, and the integral absorbance/area was used as the quantitative results of the detection index. We also quantified horizontal cell (HC) bodies by counting Calbindin-D positive cells in the fields.

For double signal labeling, immunofluorescent staining was employed. The sections underwent incubation with secondary antibodies conjugated to fluorophores at a temperature of 37 ℃ for a duration of 2 h. The nuclei were then stained using 4′,6-diamidino-2-phenylindole (DAPI, procured from Sigma-Aldrich). The immunofluorescent staining was subsequently analyzed with the aid of a Zeiss LSM 5 Pascal confocal microscope, which was connected to a Zeiss Axiovert 200 M microscope (originating from Jena, Germany). At least 3 eyes (from 3 different mice) in each group were selected, and 5 randomly selected images with 40× were captured from each retinal sample. The cells in the INL that exhibited positive staining for Calbindin D were used to quantify the HC number. The areas of PKCα and CtBP2 positive immunofluorescence were measured using ImagePro Plus 6.0 software, using the following equation: ${\text{Percent}}\;{\text{positive}}\;{\text{area}} = {\text{positive}}\;{\text{area/total}}\;{\text{tissue}}\;{\text{area}}.$

### Statistical analysis

The assessment of the data was carried out utilizing an independent two-sample *t*-test and ANOVA, employing the statistical program SPSS 26.0 (IBM Inc., Armonk, NY, USA). The results are presented as the mean value ± the standard deviation (SD). A *P*-value of less than 0.05 was considered to indicate statistical significance.

## Results

### Expression of *pde6b* mRNA and early-stage developmental histological changes in rd1 mice

**Figure 1:**
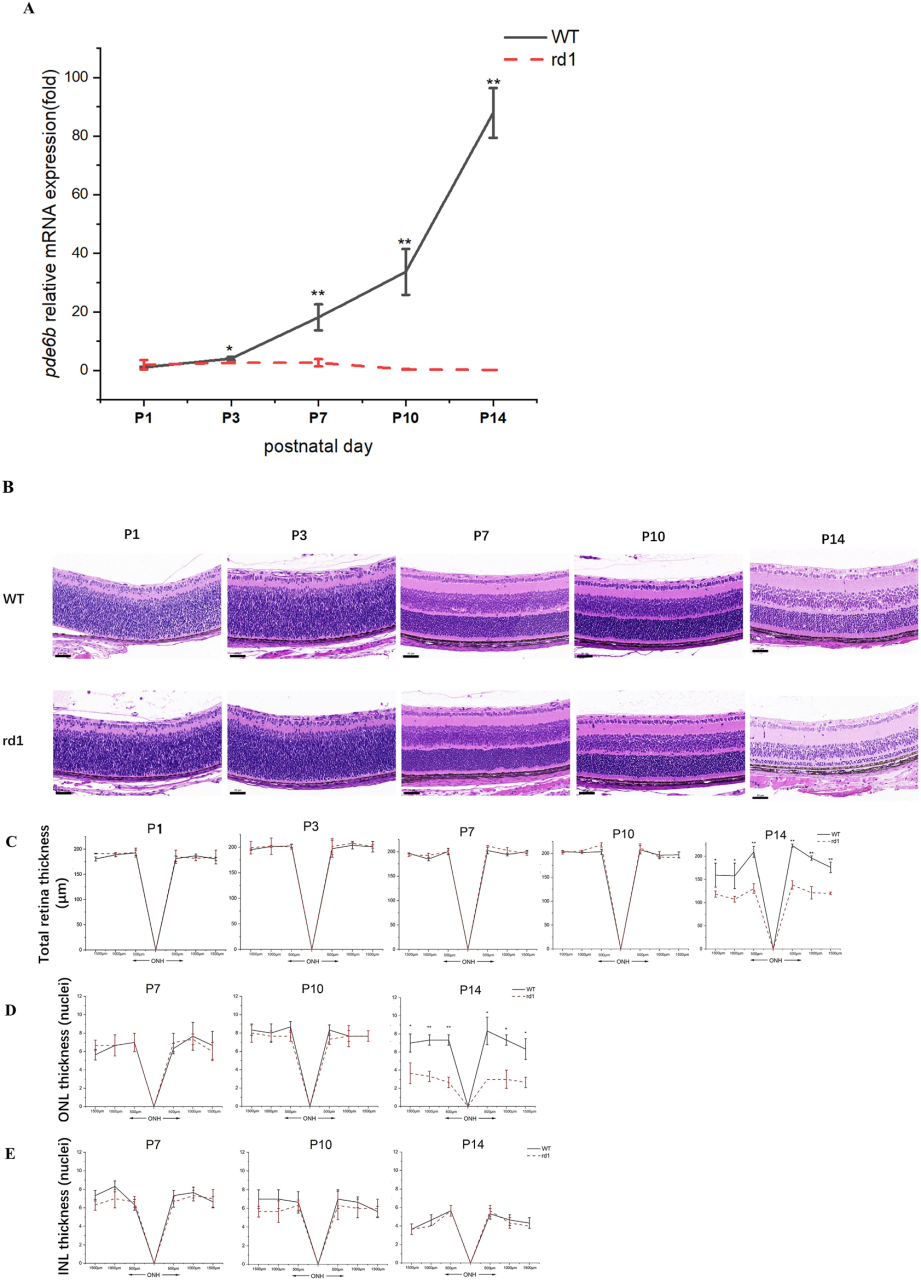
(**A**) Quantitative RT-PCR analysis of the levels of expression of *pde6b* mRNA from P1 to P14. In WT mice, *pde6b* expression gradually increased from P1 to P14. In rd1 mice, the level of *pde6b* mRNA expression was very low from P1 and showed significant differences compared with WT mice from P3. (N = 3, and each sample was pooled from four retinas from two different litters). (**B**) Histological sections of WT and rd1 mouse retinas. The ONL of rd1 mice thinned to merely a few layers at P14 compared to the WT mice's ONL. Image scale bar: 50 μm. (**C**) The thickness of total retina of WT mice and rd1 mice at the indicated positions from the optic nerve head (ONH). The total retina thickness of rd1 mice at P14 was significantly different from that of WT mice. (**D**) The thickness of outer nuclear layer (ONL) for both WT and rd1 mice. The nuclei count of the ONL of rd1 mice at P14 differed significantly from that of WT mice. (**E**) The thickness of inner nuclear layer (INL) of WT mice and rd1 mice. There was no significant difference in the nuclei count of the INL between rd1 and WT mice at different postnatal days. (N = 3 eyes from different mice, n = 3 images from each eye). **P < 0.01, *P < 0.05 compared with rd1 mice by an independent two-sample *t*-test.

To verify the gene expression trend in rd1 retina during early postnatal development, *pde6b* mRNA expression levels were examined. The relative expression of *pde6b* for WT mice at postnatal day 1 (P1), 3, 7, 10 and 14 were 1.02 ± 0.25, 4.09 ± 0.53, 18.10 ± 2.59, 33.65 ± 7.83, 87.91 ± 8.52, respectively, and the relative expression for rd1 mice were 1.91 ± 1.63, 2.63 ± 0.21, 2.59 ± 1.28, 0.36 ± 0.12, 0.14 ± 0.01, respectively (Fig. 1A). In WT mice, *pde6b* expression increased from P1 to P14. In rd1 mice, the level of mRNA expression of *pde6b* displayed low gene expression and were significantly lower compared with WT mice from P3 (Fig. 1A).

The neural retina of a newborn mouse exhibited the ganglion cell layer (GCL), inner plexiform layer (IPL), and a dense neuroblast cell layer. By P7, the outer and inner nuclear layers were fully distinct, and the OPL emerged. The histological morphology of the rd1 mouse retina from P1 to P10 was indistinguishable from that of the WT mouse retina (Fig. 1B), while the alterations in the histology of rod photoreceptor cells within the rd1 retina were initially observed around P14 (Fig. 1C,D). The entire thickness of the retina reduction was most obvious of rd1 mice retina at P14 (Fig. 1C), which had 2–4 rows of nuclei in ONL (Fig. 1D). The inner layers remained intact without any apparent loss of cells and the INL of the rd1 mice appeared less affected in retinal morphology as compared to the WT mice (Fig. 1E).

### Ultrastructural alterations in early-stage rd1 mice and their WT counterparts

**Figure 2:**
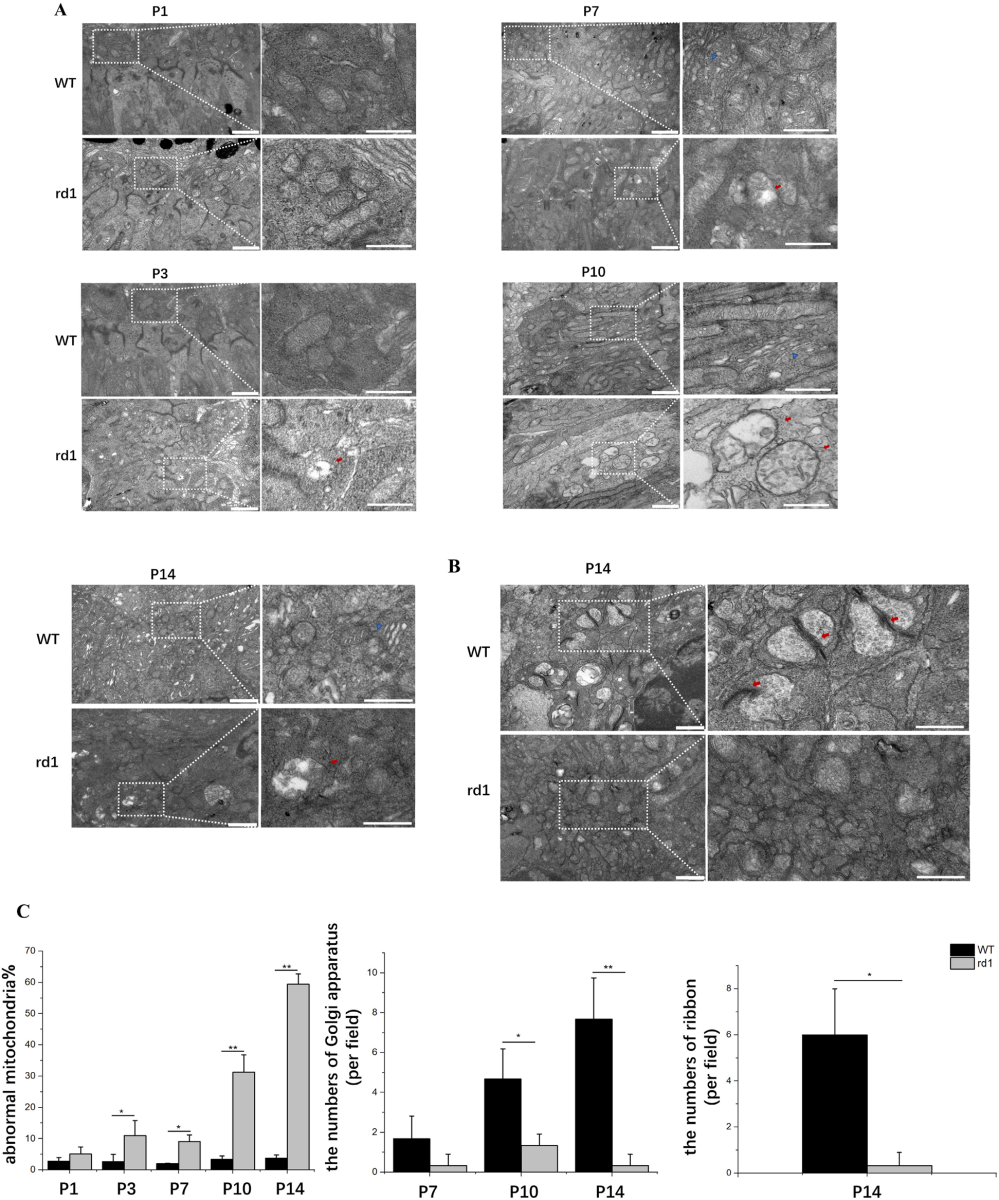
Electron micrographs of WT and rd1 mice. (**A**) Morphological defects in mitochondrial cristae structure (red arrows) at P3 in rd1 retinas. Abnormal mitochondria (red arrows) became more widespread in rd1 rod photoreceptor inner segments from P7. The Golgi apparatus (blue triangles) emerged within the inner segments at P7 and showed extensive at P14 in WT mice. In rd1 mice, the Golgi apparatus was scarcely observed from P7. Image scale bar: 1000 nm; 500 μm (magnified graphs). (**B**) Through TEM, a group of rod terminals was observed at P14 in the OPL of wild-type mice, with synaptic ribbons marked by red arrows. In the OPL of rd1 mice, the terminals contained vesicles and a possible invagination accompanied by postsynaptic structures. Image scale bar: 500 nm. (**C**) Quantification of abnormal mitochondria, the number of Golgi apparatus and ribbon indication showed that mitochondria cristae structure defected as early as P3, few Golgi apparatus were seen from P7 and few ribbons at P14 in the rd1 retina. (N = 3 eyes from 3 different mice, n = 6 images from each eye). **P < 0.01, *P < 0.05 compared with rd1 mice by an independent two-sample *t*-test.

Mitochondria accumulation was noticeable within the photoreceptor inner segments and adjacent cytoplasmic areas in both rd1 and WT mice at P1. Through the application of TEM, we observed a delay in the organization of rod inner segments and defects in the structure of mitochondrial cristae in rd1 retinas, starting at P3. By P7, there was a noticeable increase in the presence of abnormal mitochondria in the inner segments of rd1 rod photoreceptors, characterized by a loss of inner membrane cristae and a swollen, vesicular appearance. Despite these abnormalities, most mitochondria at this stage still retained their typical overall morphology. As the study progressed to P10, the swollen mitochondria in rd1 rods increased in both size and quantity, indicating a disruption in the mitochondrial network prior to the onset of observable rod cell death. By P14, extensive degeneration was evident, with only a small number of mitochondria in rd1 rods displaying normal overall or ultrastructural morphology (Fig. 2A,C).

Another notable difference was the presence of the Golgi apparatus within the inner segments at P7 and showed extensive at P14 in WT mice, while few Golgi apparatus were observed in rd1 mice from P7 onwards (Fig. 2A,C).

To investigate the impact of extensive degeneration on photoreceptor synapse ultrastructure, we examined rd1 and WT retinas at P14. In WT retinas, we detected rod photoreceptor terminals and a significant number of anchored ribbons in the OPL. Conversely, in the rd1 OPL, structures akin to synaptic terminals with membrane-bound features were present, yet the typical indentations of postsynaptic processes within the terminal were absent (Fig. 2B,C).

### Early-stage synaptic interactions in rd1 retina involving rod bipolar cells (RBCs) and horizontal cells (HCs) in the OPL

In the retinas of WT mice, second-order neurons, such as RBCs identified by PKCα antibodies, and HCs marked by calbindin D28K antibodies, form synapses with photoreceptor terminals in the OPL. At the site of rod synapses, a single presynaptic ribbon is situated next to postsynaptic processes of HCs and RBC dendrites, which extend into a photoreceptor terminal invagination^26^.

**Figure 3:**
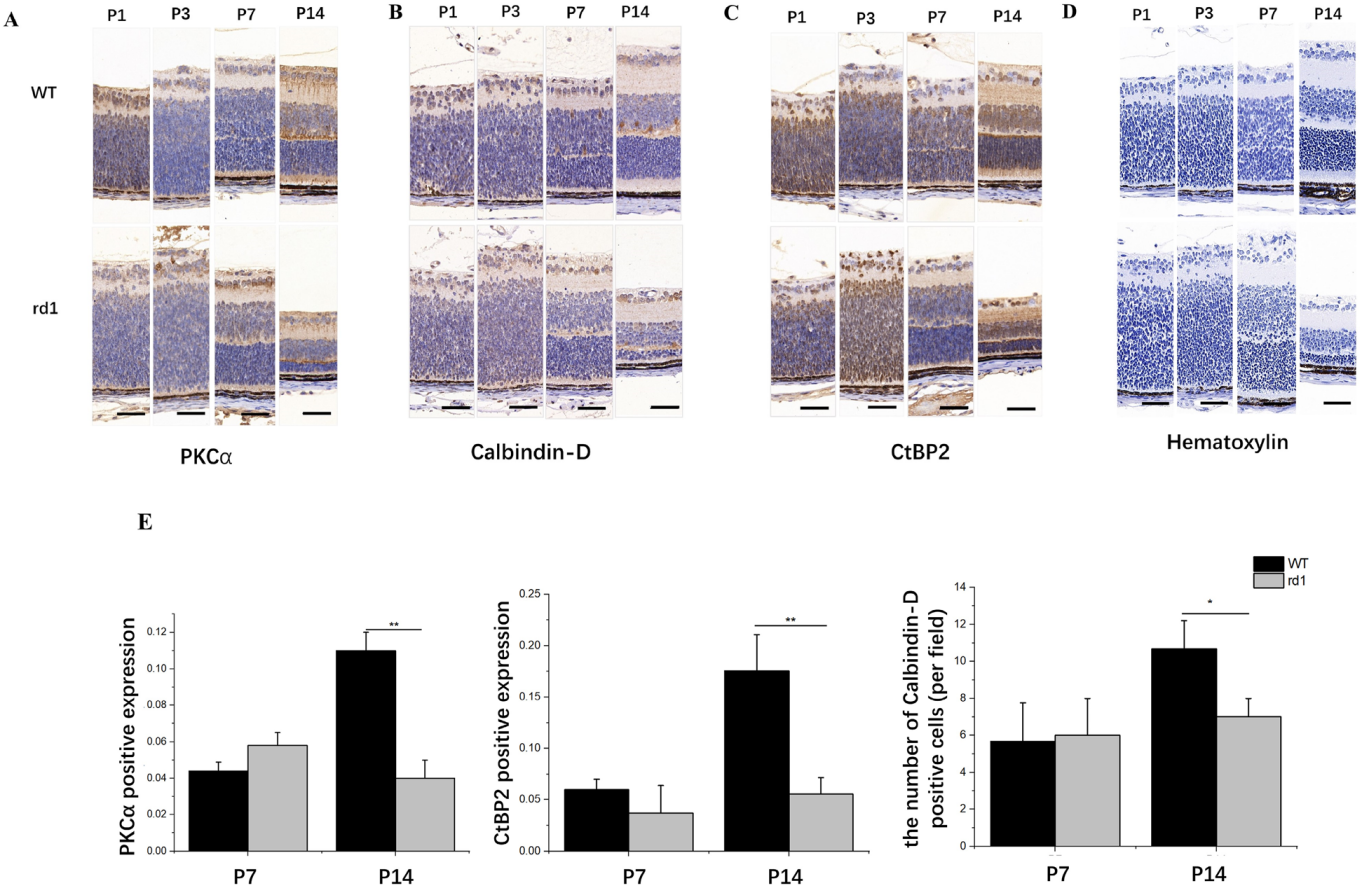
PKCα staining of rod bipolar cells (**A**), Calbindin staining of horizontal cell bodies (**B**), CtBP2 staining of presynaptic ribbon (**C**), hematoxylin staining of the nuclei (**D**) in WT and rd1 mouse retinal sections, and Quantification of immunohistochemical staining (**E**). No noticeable differences were observed in the staining of rd1 and WT retinas at P7. At P14, immunoreactivity for PKCα and CtBP2 in the OPL of the rd1 retina appeared reduced, and a reduction in processes originating from horizontal cell bodies was also observed. Image scale bar: 50 μm (N = 3 eyes from different mice, n = 5 images from each eye). **P < 0.01, *P < 0.05 compared with rd1 mice by an independent two-sample *t*-test.

The presynaptic ribbon was marked with the RIBEYE marker using the CtBP2 antibody. As the OPL rudiments were present at P7, synaptic connections can be observed from this time point. Immunohistochemical differences between WT and rd1 mice were barely discernible before P7 (Fig. 3). Significant synaptic alterations occurred at P14 in rd1 mice, with RBCs being severely impacted by photoreceptor degeneration (Fig. 3A,E). Similarly, the number of HC bodies slightly decreased (Fig. 3B,E), and the quantity of CtBP2-positive puncta significantly declined in the retinas of rd1 mice at P14 compared to WT mice (Fig. 3C,E).

**Figure 4:**
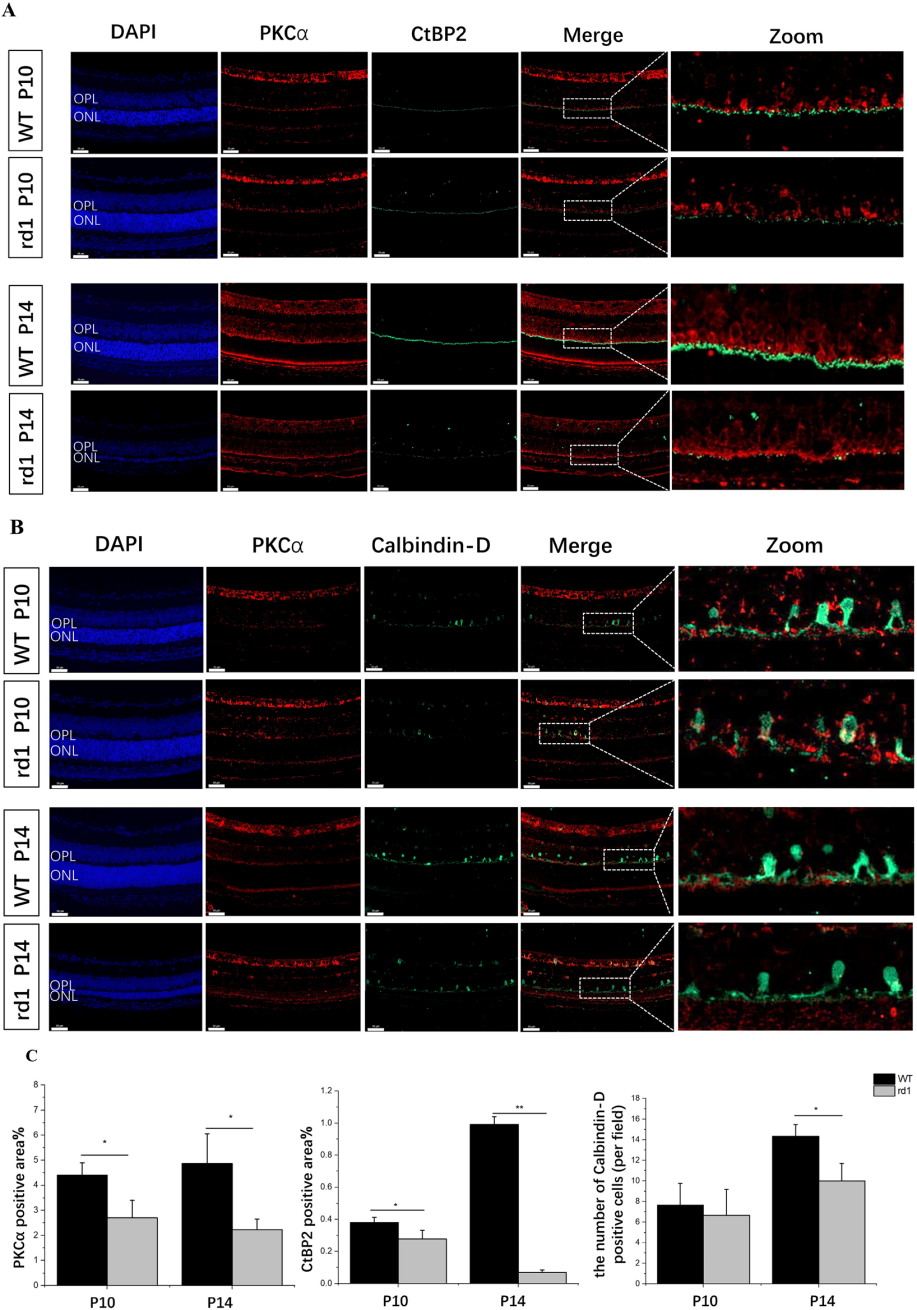
(**A**) Retina double labeling with PKCα (rod bipolar cells, red) and CtBP2 (presynaptic ribbon, green) antibodies revealed that CtBP2-positive puncta were sparse and appeared flattened in rd1 retina from P10, before photoreceptor degeneration. (**B**) In WT and rd1 retinas with both RBCs (labeled with PKCα in red) and HCs (marked with Calbindin in green) identified, the terminal dendrites of horizontal cells in the rd1 retina experienced a reduction since P10, and horizontal cell bodies decreased at P14. Image scale bar: 50 μm. OPL, outer plexiform layer; ONL, outer nuclear layer. (**C**) Quantification of immunofluorescence staining indicated the reduction of CtBP2 and PKCα positive area during retina degeneration and the significant loss of horizontal cell bodies per field at P14. (N = 3 eyes from different mice, n = 5 images from each eye). **P < 0.01, *P < 0.05 compared with rd1 mice by an independent two-sample *t*-test.

To investigate synaptic protein expression patterns in the developing rd1 retina, immunofluorescence staining was carried out using antibodies raised and colocalized puncta during P10–P14 (Fig. 4). Dense rod bipolar dendrites were visible in the OPL up to P14 but were sparse and flattened in the rd1 retina affected by photoreceptor degeneration. CtBP2 showed an abnormal distribution from P10, preceding photoreceptor degeneration (Fig. 4A,C). By P14, the CtBP2 band displayed a notable reduction, resulting in a singular layer characterized by occasional, dispersed spots (Fig. 4A). HC bodies maintained their normal morphology but decreased at P14 in the rd1 retina (Fig. 4B,C); however, terminal dendrites decreased starting from P10 (Fig. 4B). Dendritic loss was not unexpected in neurons that lost their afferent synapses. Our findings suggested that the primary morphological and quantitative synaptic changes in the OPL occurred in rd1 mice at P10.

### Prolonged preservation of photoreceptor cells and sustained visual function in rd1 mice following oral administration of idebenone

**Figure 5:**
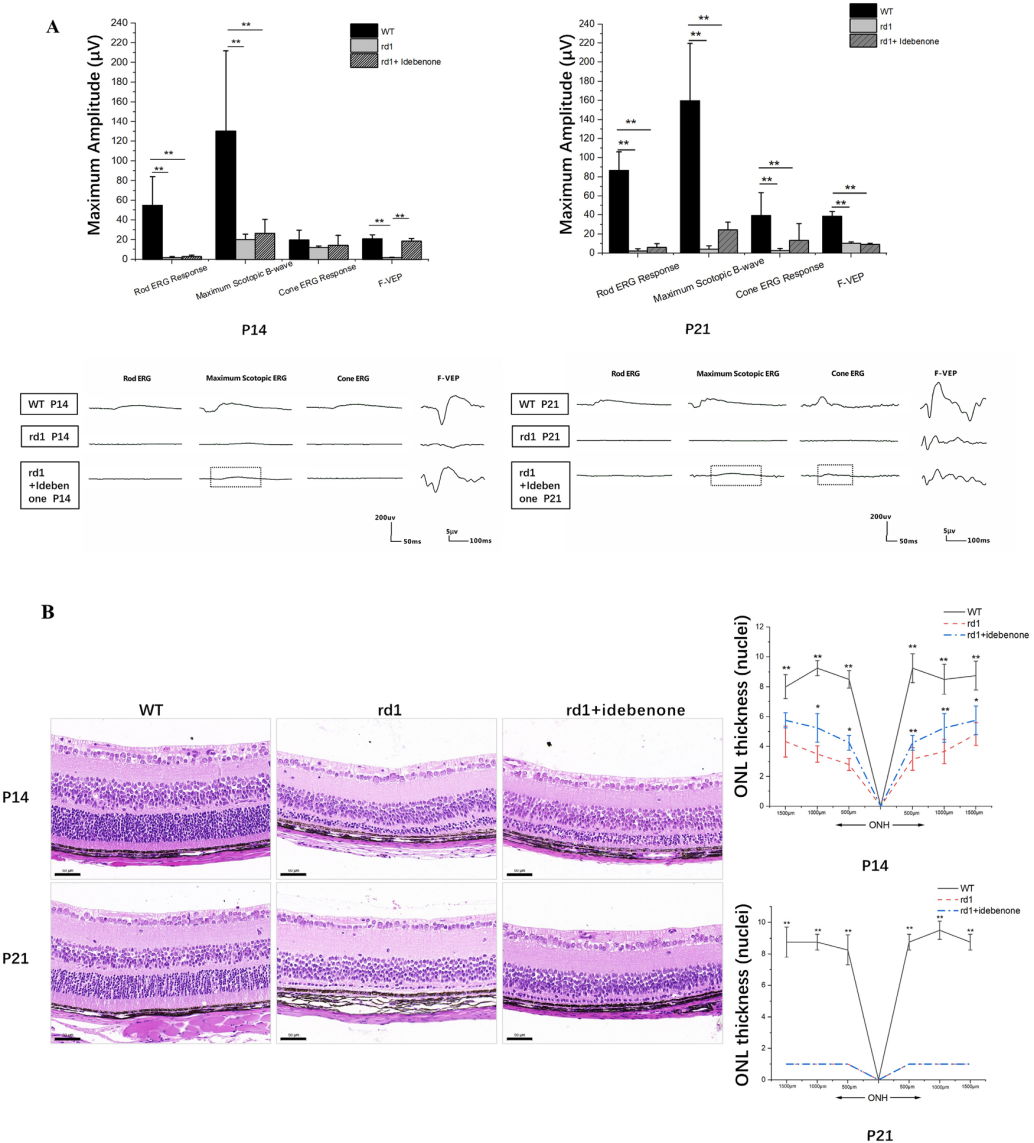

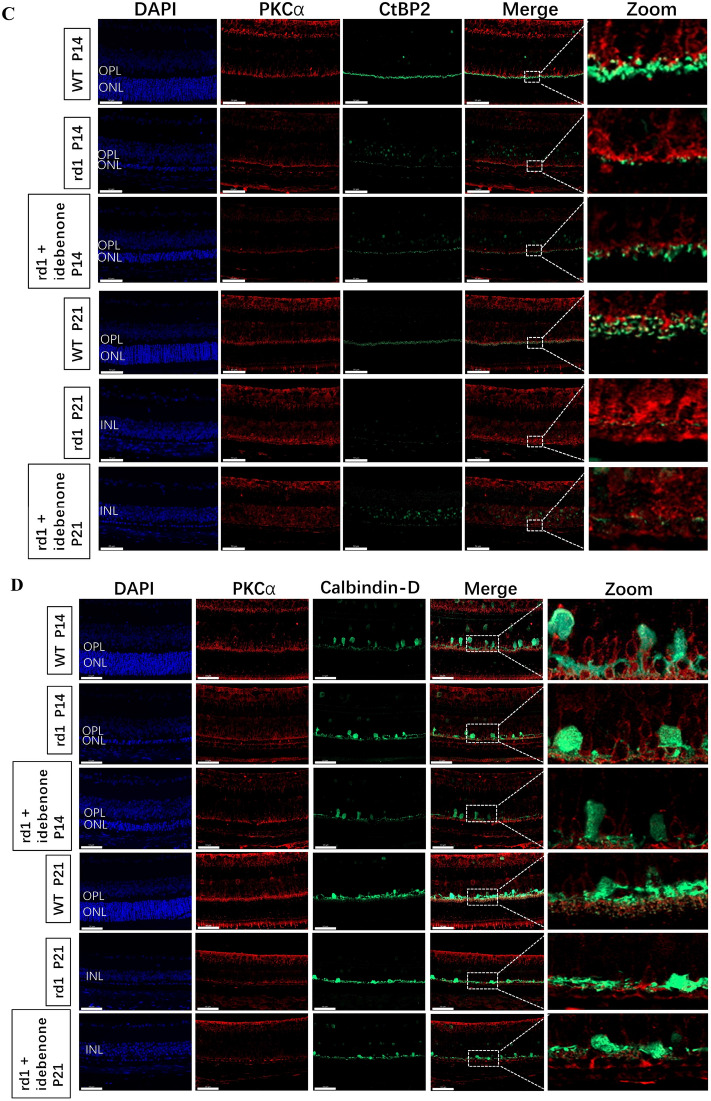

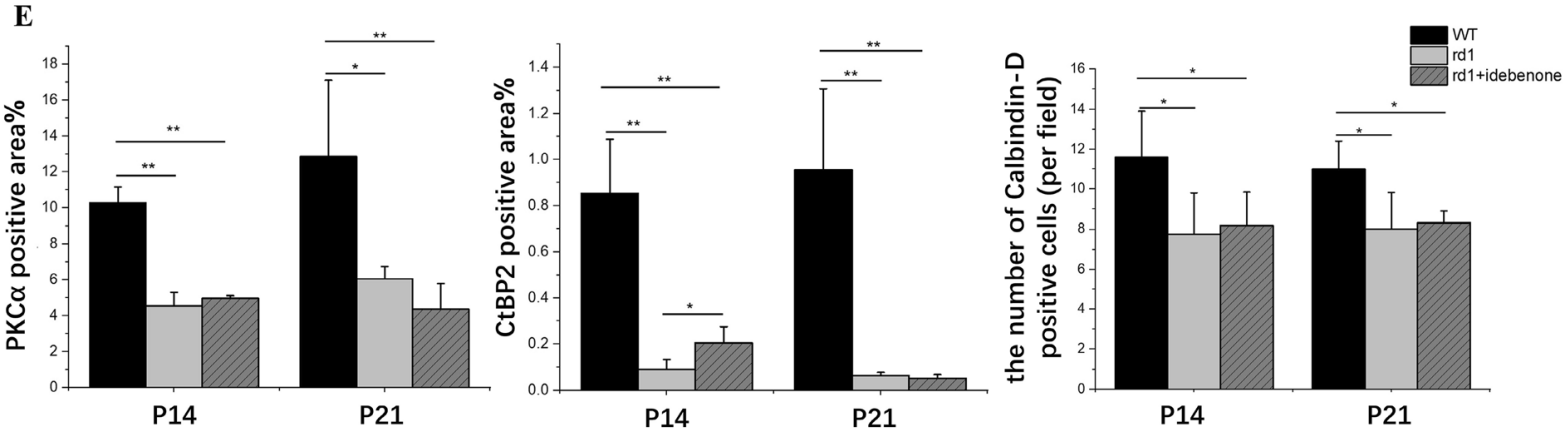
Idebenone supplementation delayed photoreceptor cell death in rd1 retinas. (**A**) Quantification of ERG indicated that b-wave amplitudes of ERG showed no significant visual rescue in mice treated with idebenone, although there was a trend toward an increased response (black square box). WT mice exhibited more distinct waveforms than rd1 mice, irrespective of treatment. However, the amplitude of F-VEP showed significant visual rescue in mice treated with idebenone at P14, which was not significant at P21. (N = 4–10 eyes from different mice). (**B**) Histological examination of untreated or idebenone-treated rd1 retinas revealed that idebenone therapy promoted neuronal cell survival in the ONL at P14, though the effect was not significant at P21. Morphometric analysis of ONL thickness extending from the ONH also indicated that idebenone treatment notably preserved photoreceptor cells at P14. (N = 4–10 eyes from different mice, with multiple counts per eye). Image scale bar: 50 μm. (**C**) Retinal double staining using antibodies against PKCα (red) and CtBP2 (green) showed an increase in CtBP2-positive puncta following enhanced photoreceptor cell survival in rd1 mice treated with idebenone for 14 days, while the numbers remained unaltered and scarce in the treated groups at P21. In WT mice, the characteristic horseshoe-shaped CtBP2-positive puncta were observed, particularly at P21. (**D**) Rod bipolar cell (labeled with PKCα, red) and horizontal cell (marked by Calbindin, green) double-labeling revealed a substantial decrease in the terminal dendrites of horizontal cells in rd1 mice as opposed to their WT counterparts, while the untreated and idebenone-treated mice at the ages of P14 and P21 showed no differences. INL, inner nuclear layer; ONH, optic nerve head. Image scale bar: 50 μm. (**E**) Quantification of immunofluorescence staining indicated only CtBP2-positive area were increased following the photoreceptor cell survival in the rd1 mice after idebenone treatment for 14 days. (N = 3–6 eyes from different mice, n = 5 images from each eye). **P < 0.01, *P < 0.05 compared with untreated rd1 and idebenone-treated rd1 mice by one-way ANOVA with LSD’s multiple comparisons.

As mentioned earlier, we observed mitochondrial damage as early as P3. Idebenone, a synthetic quinone analog to coenzyme Q10 (CoQ10), protects mitochondria from oxidative damage^27^. We hypothesized that idebenone treatment could extend photoreceptor cell viability in retinal degeneration by enhancing mitochondrial activity. To investigate this, nursing mother mice were given a daily dose of idebenone via gavage starting on postpartum day 0. Mice administered idebenone displayed no noticeable issues such as reduced appetite, infection, or impaired motor function. We analyzed the early-stage progression of the disease in littermates at P14 and P21 (Fig. 5).

Our findings revealed that idebenone-treated rd1 mice exhibited increased b-wave scotopic and photopic ERG responses compared to untreated rd1 mice, though the differences were not significant (Fig. 5A). Comparatively, the amplitude of VEP is believed to be closely related to the transmission of visual axonal signals. Therefore, we subjected the mice to F-VEP testing. The results showed idebenone-treated rd1 mice had an increase in amplitude at P14, indicating prolonged visual function (Fig. 5A). At P14, histological examination of the retina demonstrated that treatment with idebenone notably improved the survival of neuronal cells within the ONL, but by P21, this efficacy of the treatment in rescuing photoreceptor cells diminished (Fig. 5B). Double-labeled synaptic immunocytochemistry was employed to visualize surviving second-order synaptogenesis. RBC levels remained largely unchanged, but the proportion of CtBP2-positive puncta notably increased in the rd1 mice after 14 days of idebenone treatment, while the numbers remained unaltered and sparse in the treated groups at P21 (Fig. 5C,E). In WT mice, the characteristic horseshoe-shaped CtBP2-positive puncta were particularly evident at P21 (Fig. 5C). In rd1 mice, there was a notable decrease in the terminal dendrites of horizontal cells when compared to their WT counterparts. No observable distinctions were found between untreated mice and those administered with idebenone at P14 and P21 time points (as shown in Fig. 5D,E).

## Discussion

Mammalian retinas notably lack the capacity for regeneration, which leads to photoreceptor cell malfunction and/or demise, serving as a primary cause of incurable vision loss. Abnormal retinas, when juxtaposed against their healthy counterparts, present developmental variations. These variations can be classified into three distinct categories: predegenerative differences observed before any clear signs of degeneration, degenerative changes leading to photoreceptor cell dysfunction, and postdegenerative effects that manifest near the conclusion of photoreceptor degeneration, primarily affecting the remaining retinal layers^28^. Our investigation focused on the early stages before significant photoreceptor loss. We propose that genetic changes could alter molecular and biochemical processes within target cells during the initial stages of retinal development. Our aim was to pinpoint an optimal treatment window to delay photoreceptor cell death.

The rd1 mouse is a universally recognized model for retinal degeneration research. *Pde6b* plays a crucial role in controlling cGMP levels, and a buildup of cGMP has been linked to the death of rod photoreceptors in rd1 retinas, as well as to inherited retinal diseases connected to phototransduction^29^. We previously reported that almost no PCR products were detected for *pde6b* gene mRNA expression in rd1 at P14^22^. In this study, we observed a gradual increase in *pde6b* expression in WT mice from P1, but it was notably low during early postnatal development in rd1 mice. Remarkably, photoreceptor cell death in the rd1 retina begins around P14, which corresponds to the mouse's eye-opening time. *Pde6b* may serve an underexplored function in photoreceptor development, separate from its role in phototransduction in mature retinas, potentially leading to the initiation of cell death. During various developmental stages, cells undergo a specific differentiation sequence. Differentiation begins with retinal ganglion cells (RGCs), followed by horizontal and amacrine cells. Subsequently, rods and bipolar cells develop, culminating in Müller cell emergence^30,31^. The development of inner retinal layers continues as distinct retinal neuron categories mature throughout postnatal development^31^. While no histological differences were observed between the retinas from birth to P10, our ultrastructural analysis identified mitochondrial damage in developing rd1 rods as early as P3, in line with a prior report^29^. Photoreceptors demand significant energy to maintain their physiological state both in light and darkness^32^, with mitochondria pivotal in fulfilling these energy needs. Proteomic and metabolomic analyses of P6 rd1 retinas indicate that mitochondrial processes, coupled with related metabolic and calcium signaling pathways, are integral during the initial cellular responses in retinal neurodegeneration^29^. Crucially, no substantial variation in cGMP levels was observed between the metabolomic data from P6 rd1 and WT retinas^29^. We noted a significant difference in the *pde6b* mRNA expression levels in rd1 mice compared to WT mice from P3 onwards. Identifying early patterns related to the disease, as shown in this study, underscores the critical role of mitochondria in the cell's response to genetic changes, potentially influencing events before neurodegeneration onset. We suggest a connection between the rd1-specific *pde6b* mutation and mitochondria that predates the physiological regulation of cGMP channels. This connection likely stems from an impaired energy balance and atypical metabolic and calcium signaling processes^29^.

In the normal retina, the appearance of the Golgi apparatus inside the inner segments was P6 ~ 8^28^. Few Golgi apparatus were observed in rd1 mice from P7, a finding similarly observed by Sanyal and Bal^28^ at 6 days. As intricate ciliated sensory neurons, photoreceptors rely on the collaboration between the basal body, periciliary ridge, and Golgi complex for the regulation of protein transport from the inner to the outer segment of sensory axonemes^33^. In the mutant retina, the growth of Golgi elements and outer segments occurred concurrently^28^. The Prenylated Rab Acceptor 1 (PRA1/Rabac1) is an essential four-pass transmembrane protein situated in the Golgi apparatus and plays a crucial role in the process of protein trafficking. It was found to be significantly downregulated in the rd1 mouse retina at P2^34^ and was inherently located within the endoplasmic reticulum (ER), specifically at the membrane contact sites between the ER and mitochondria^35^. We propose that organelles such as the mitochondria, Golgi apparatus, and endoplasmic reticulum may have reciprocal influences. Mitochondrial degradation affects the development of the Golgi apparatus, subsequently leading to dysplastic outer segments.

The development of retinal gene therapy techniques has made gene replacement a viable option for patients with certain inherited retinal degenerations. Researchers are now focusing on expanding the application of these vectors using alternative strategies, like optogenetics–the introducing of exogenous light-sensitive proteins into excitable cells^36^. The underlying assumption of this method is that the functional characteristics and connections of inner retinal neurons are preserved even after the degeneration of photoreceptors^13^. An electron microscopy study revealed that between P7 and P14, bipolar cell dendrites invaginate into dyads, forming triads and completing the photoreceptor ribbon synapse^37^. We conducted a developmental study on the rd1 mouse's inner retina, demonstrating that despite their normal morphological presentation, the second-order neurons in the rd1 retina never establish normal synaptic contacts, a finding similarly observed by Blanks et al.^37^. The discovery indicates that anomalous synaptogenesis occurring at the initial synapse of the visual pathway is a prevalent feature among phenotypes caused by mutations in rod-specific genes, such as *pde6b*^7,38^. Our findings further corroborate the idea that a solitary genetic alteration, even when restricted to a specific cell type, can exert a widespread impact on the retina as well as other neuronal populations. Significant retinal remodeling after photoreceptor demise^36,39^, and gene expression changes associated with synaptic remodeling, neurotransmitter release, and viral vector entry are also noted in ON-bipolar cells during degeneration^36^. The results of this study suggest that emphasize the importance of into account the considerable impact on inner retinal neurons when undertaking neural reconstruction in retinas experiencing degeneration.

In September 2015, Idebenone, an analog of CoQ10 and short-chain ubiquinone, received approval in the European Union for the treatment of Leber's Hereditary Optic Neuropathy (LHON), a disorder linked to dysfunctional complex I subunits in the mitochondrial electron transport chain^40^. We employed doses within the clinical therapeutic range for LHON (900 mg/day)^40^, adjusted for mice, taking into account their body surface area. Our study revealed that oral administration of idebenone extended photoreceptor cell survival and enhanced retinal second-order synaptogenesis in rd1 mice at P14. The retina is composed of a complex and meticulously arranged network of neuronal cells, which are interconnected through synapses. Given the high genetic heterogeneity of retinal degenerative diseases, alterations in different genes may lead to identical clinical manifestations of a disease, suggesting that shared mechanisms exist for photoreceptor degeneration, independent of the causative genetic mutation^41^. Additionally, recent studies have uncovered the significant impact of mitochondrial dysfunction in the development of age-related neurodegenerative disorders, including age-related macular dystrophy, Alzheimer's disease, Parkinson's disease, and Huntington's disease^41^. Rowe et al. described a 'metabolic ecosystem' in the retina of a 'metabolic ecosystem' in the retina, with dietary supplementation of tricarboxylic acid (TCA) cycle intermediates demonstrating a reduction in the rate of disease progression in *Pde6α* and *RhoP23H* mouse models of retinal degeneration^41^. A recent study by Yang J et al. demonstrated that fullerenols can effectively alleviate photoreceptor degeneration in rd1 mice by reversing the abnormal mitochondrial DNA and nuclear DNA expression patterns of the electron transport chain and restoring mitochondrial function in degenerating photoreceptors^42^. However, when we explored the effects of idebenone supplementation of idebenone supplementation during a middle phase of the disease (P21) and found no substantial enhancement in visual acuity. Davis RJ et al. also showed that a tamoxifen inducible Cre-loxP rescue allele, *Pde6b*^*Stop*^, at later time-points (P1) indicated partial long-term or short-lived rescue and suggested that complete, partial, and incomplete restoration of retinal function depend on the number of treated photoreceptors^43^. The reason may lie in the fact that, at this stage (P21), the retina consisted of a single layer of photoreceptor cell nuclei, lacking inner segments containing mitochondria. This could potentially render it unable to support deteriorating rod photoreceptor neurons by increasing mitochondrial activity. Alternatively, it is possible that various metabolic pathways or other cells, such as RPE and Müller glia, within the neural retina, may provide support to photoreceptors at distinct developmental stages.

## Conclusions

While idebenone is clinically employed to treat mitochondrial-related hereditary optic atrophy, its dosage and administration timing require further examination is necessary before considering it as a therapeutic option for retinitis pigmentosa. Indeed, we analyzed mitochondrial morphology after idebenone treatment and found no significant changes (data not shown). This study aimed to explore the mechanisms underlying early-stage photoreceptor cell deterioration in their initial stage of disease in vivo, rather than offering an immediate treatment solution. Our findings present a mechanistic framework indicating that mitochondrial damage serves as an early driver of photoreceptor cell death in retinal degeneration.

## Data Availability

The datasets used and/or analysed during the current study available from the corresponding author on reasonable request.
